# Meta-analysis of the diagnostic value of functional magnetic resonance imaging for distinguishing unresponsive wakefulness syndrome/vegetative state and minimally conscious state

**DOI:** 10.3389/fnins.2024.1395639

**Published:** 2024-09-09

**Authors:** Helin Zheng, Lu Tian, Jinhua Cai

**Affiliations:** Department of Radiology, Ministry of Education, Key Laboratory of Child Development and Disorders. Chongqing Key Laboratory of Pediatrics, Children’s Hospital of Chongqing Medical University, National Clinical Research Center for Child Health and Disorders, Chongqing, China

**Keywords:** fuctional magenetic resonance imaging, unresponsive wakefulness syndrome/vegetative state, minimally conscious state, differential diagnosis, meta-analysis

## Abstract

**Objective:**

Unresponsive wakefulness syndrome/vegetative state (UWS/*VS*) and minimally conscious state (MCS) are considered different clinical entities, but their differential diagnosis remains challenging. As a potential clinical tool, functional magnetic resonance imaging (fMRI) could detect residual awareness without the need for the patients’ actual motor responses. This study aimed to investigate the diagnostic value of fMRI for distinguishing between UWS/*VS* and MCS through a meta-analysis of the existing studies.

**Methods:**

We conducted a comprehensive search (from the database creation date to November. 2023) for relevant English articles on fMRI for the differential diagnosis of UWS/*VS* and MCS. The pooled sensitivity, specificity, positive likelihood ratio (LR+), negative likelihood ratio (LR−), summary receiver operating characteristic (SROC) curve, and area under the curve (AUC) were calculated to assess the diagnostic value of fMRI in distinguishing between UWS/*VS* and MCS. The statistical *I*^2^ test was used to assess heterogeneity, and the source of heterogeneity was investigated by performing a meta-regression analysis. Publication bias was assessed using the Deeks funnel plot asymmetry test.

**Results:**

Ten studies were included in the meta-analysis. The pooled sensitivity and specificity were 0.71 (95% CI 0.62–0.79) and 0.71 (95% CI 0.54–0.84), respectively. The fMRI for the differential diagnosis of UWS/*VS* and MCS has a moderate positive likelihood ratio (2.5) and a relatively low negative likelihood ratio (0.40). Additionally, SROC curves showed that the AUC was 0.76 (95% CI 0.72–0.80).

**Conclusion:**

Functional magnetic resonance imaging has a good performance in the differential diagnosis of UWS/*VS* and MCS, and may provide a potential tool for evaluating the prognosis and guiding the rehabilitation therapy in patients with disorders of consciousness.

## 1 Introduction

The unresponsive wakefulness syndrome/vegetative state (UWS/*VS*) and minimally conscious state (MCS) are recognized as distinct clinical entities within the spectrum of disorders of consciousness (DoC) (Ferre et al., 2023; Yang et al., 2023; Porcaro et al., 2022). UWS/*VS* is a clinical condition characterized by wakefulness with the opening of eyes, but remaining unawareness and non-reflexive, non-purposeful behavioral responses (Giacino et al., 2014). MCS is a neurological condition in which individuals may exhibit minimal, inconsistent but replicable voluntary behaviors, indicating more signs of consciousness compared to those with UWS/*VS* (Giacino et al., 2014). The etiology of these conditions can vary, encompassing traumatic brain injury, stroke, anoxic brain injury, neurodegenerative disease, and so on. Among them, traumatic brain injury is one of the most common observed causes (Sun et al., 2020). The prevalence of DoC is fully unknown in many countries, showing significant variability due to various factors such as geographic areas, diagnostic and case definition criteria, the setting for case ascertainment, and so on (Pisa et al., 2014; van Erp et al., 2015). Pisa et al. (2014) identified that prevalence ranged from 0.2 to 3.4 cases per 100,000 inhabitants for *VS* and 1.5 per 100,000 for MCS in a systematic review. In a nationwide point prevalence study conducted by van Erp et al. (2015), in the Netherlands in 2015, the prevalence of hospitalized and institutionalized *VS*/UWS patients in the general population was estimated at 0.1–0.2 per 100,000 individuals.

Generally, individuals with MCS tend to have better prognosis and respond more favorably to rehabilitative treatments than those with UWS/*VS* (Giacino et al., 2014). Family members and healthcare providers are more inclined to actively treat and rehabilitate individuals with a higher likelihood of regaining consciousness, as opposed to those individuals with a lower probability of recovering consciousness (Giacino et al., 2014). Therefore, accurate differentiation between the two conditions has significant ethical and practical implications in the clinical management of individuals with DoC.

The conventional assessment tools primarily encompass clinical behavior scales based on bedside neurobehavioral measurements (Formisano et al., 2019; Formisano et al., 2018; Cortese et al., 2023). However, these clinical scale evaluations lack precision and objectivity in providing diagnostic insights for individuals with DoC. Despite the Coma Recovery Scale-Revised (CRS-R) being acknowledged as the most reliable and valid clinical tool, underestimation of levels of consciousness might occur in 37–43% of DoC patients (van Erp et al., 2015; Annen et al., 2019; Schnakers et al., 2009; Jain and Ramakrishnan, 2020; Kondziella et al., 2020). In addition to behavioral scales, various neuroimaging and electrophysiological techniques play crucial roles in distinguishing between UWS/*VS* and MCS (Porcaro et al., 2022; Paunet et al., 2023; Sebastiano et al., 2021). For example, positron emission tomography (PET) provides quantitative information about brain metabolic activity, aiding in the assessment of consciousness levels (Paunet et al., 2023). Electroencephalography (EEG) offers real-time recording of brain electrical activity, serving as a cost-effective method to monitor potential consciousness, particularly in unresponsive states (Sebastiano et al., 2021). However, even when behavioral scales are combined with neuroimaging and electrophysiological techniques, there remains an approximate misdiagnosis rate of 15% (Jain and Ramakrishnan, 2020). This high rate can be attributed to factors such as cognitive impairment (e.g., aphasia, apraxia) and/or sensory impairment (e.g., blindness, deafness). In such cases, the absence of responsiveness does not necessarily indicate a lack of consciousness. Therefore, there is an urgent need to utilize motor-independent technologies to distinguish between individuals in these two states.

In recent years, fMRI has emerged as a promising non-invasive technique to distinguish between patients with UWS/*VS* and MCS (Yang et al., 2023; Cao et al., 2021; Marino et al., 2017), as illustrated in Appendix 1. fMRI applications in DoC primarily involve two modalities: task-based fMRI and resting-state fMRI (rs-fMRI). Task-based fMRI records brain activity during specific tasks, such as mental imagery, speech processing, counting, and calculation. Conversely, rs-fMRI captures brain activity in a “resting” state without tasks. A landmark 2006 study by Owen et al. (2006) demonstrated significant brain activation in a 23-year-old UWS/*VS* patient during a mental imagery task, comparable to that of healthy controls, indicating fMRI’s potential to detect consciousness. Rs-fMRI focuses on resting-state networks (RSN), highlighting differences in RSN activity patterns between UWS/*VS* and MCS patients. Weakened RSN connectivity correlates with consciousness loss, potentially aiding in differentiation between these states (Wang et al., 2022; Chen et al., 2018). However, fMRI studies have shown varying sensitivity (0.42–0.89) and specificity (0.20–0.96) in distinguishing UWS/*VS* from MCS (Vogel et al., 2013; Stender et al., 2014; Pipemo et al., 2012; Demertzi et al., 2014; Yu et al., 2021; Wang et al., 2019; Coleman et al., 2007; Kotchoubey et al., 2014; Crone et al., 2011; Monti et al., 2010), leading to inconclusive diagnostic utility.

In this study, we explore the diagnostic accuracy of fMRI in distinguishing between UWS/*VS* and MCS through a meta-analysis of the existing studies. Additionally, our findings provide robust evidence supporting the application of fMRI in the field of neuroimaging, particularly in dealing with the differentiation of UWS/*VS* and MCS. Moreover, we highlight the potential of fMRI as a diagnostic tool for distinguishing between different states of consciousness and guiding clinical decision-making.

## 2 Methods

This meta-analysis was conducted according to the Cochrane Handbook for Systematic Reviews of Diagnostic Test Accuracy and the Preferred Reporting Items for Systematic Reviews and Meta-Analyses (PRISMA) guidelines (Cacciamani et al., 2023).

### 2.1 Search strategy

A comprehensive search was performed using PubMed, EMBASE, the Cochrane Library, Wiley Online Library, and the Web of Science to identify relevant articles in English (database creation dated to November 2023). The search followed the population, intervention, comparison, outcome, and study design (PICOS) principle (Amir-Behghadami and Janati, 2020) (P: “UWS/*VS*, MCS,” I: “fMRI,” S: “diagnostic test”). The search employed a blend of medical subject heading (MeSH) terms and free-text terms, as follows: (“unresponsive wakefulness syndrome” [MeSH] “vegetative state” [text], or “UWS” [text], or “*VS*” [text]) or (“minimally conscious state” [MeSH] or “MCS” [text])and (“functional magnetic resonance imaging” [MeSH] or “fMRI” [text] or “functional MRI” [text]) and (“sensitivity and specificity” [MeSH] or predict* [text] or diagnose* [text] or accura* [text]). The complete PubMed search strategy is provided in Appendix 2.

### 2.2 Selection criteria

All studies utilizing task-based or resting-state fMRI to differentiate UWS/*VS* and MCS were considered eligible for inclusion. Furthermore, the studies from which a 2 × 2 table could be constructed for true positive (TP), false positive (FP), true negative (TN), and false negative (FN) values were included. Studies were excluded if they lacked an explicitly stated reference standard, or if there were insufficient data to calculate the study outcomes. Animal experiments, case reports, meta-analyses, and reviews were excluded from this study.

### 2.3 Study selection and data extraction

The study titles and abstracts that met the inclusion criteria were screened before full-text review. Two researchers were assigned to extract data independently. Disagreements were resolved by mutual consultation, or in the absence of consensus, by discussion with a third expert. The extracted data include author, year, paradigm/method, etiology, diagnostic criteria, numbers, age, and genders of UWS/*VS* patients and MCS patients, and results (accuracy, sensitivity, and specificity). We contacted the corresponding authors of the articles when more information was required. If the reminder was not responded to, the article was deleted.

The extracted fMRI data includes: (1) data acquisition: including the brand and model of the MRI equipment, the name and version of the MRI data processing software, the sequences used (e.g., Bold sequence), and MRI equipment parameter settings (such as repetition time, echo time, and flip angle), etc.; (2) data preprocessing: including removal of motion artifacts, time correction, spatial normalization, and spatial smoothing, etc.; (3) fMRI methods: including resting-state or task-based, task design, and stimulus paradigm, etc.; (4) data quantification and analysis: data regarding the accuracy, sensitivity, and specificity of fMRI for distinguishing between UWS/*VS* and MCS were extracted from selected studies. Subsequently, we indirectly calculated metrics such as true TP, FP, TN, and FN. Finally, we conducted a meta-analysis using metrics such as TP, FP, TN, and FN. Please refer to Appendix 3 for details regarding fMRI in the included studies.

### 2.4 Quality of the studies

Individual studies were assessed for risk of bias according to the Quality Assessment of Diagnostic Accuracy Studies-2 (QUADAS-2) checklist (Qu et al., 2018) (Appendix 4). This checklist comprises 14 items divided into four parts: patient selection, index test, reference standard, and flow and timing. Within the 166-patient selection section, we controlled for possible sample overlap by carefully reviewing the sample information and basic characteristics of patients in each study. This ensured that each study’s sample was independent and non-overlapping when summarizing the data. Each article was independently evaluated by the reviewers using these criteria, and disagreements were resolved through discussion.

### 2.5 Statistical analysis

Stata software (version 20; Stata Corporation, CollegeStation, Texas, United States) was used to draw graphs and perform some calculations. Pooled sensitivity (SEN), pooled specificity (SPE), positive likelihood ratio (LR+), negative likelihood ratio (LR−), diagnostic odds ratio (DOR) with 95% confidence interval (CI), summary receiver operating characteristic (SROC) curve, and area under the curve (AUC) were calculated for diagnostic accuracy of fMRI for distinguishing between UWS/*VS* and MCS. Pooling was performed using a binary generalized linear mixture model. Heterogeneity was assessed by the chi-square test and Cochran *Q* test. If *I*^2^ > 50%, substantial heterogeneity was considered. When documenting heterogeneity between studies, potential sources of heterogeneity were explored through subgroup analyses and meta-regression analyses. Deeks’ funnel plot asymmetry test was used to check for publication bias in all included studies, and publication bias was considered significant if *p* < 0.05.

## 3 Results

### 3.1 Literature search results

**Figure 1:**
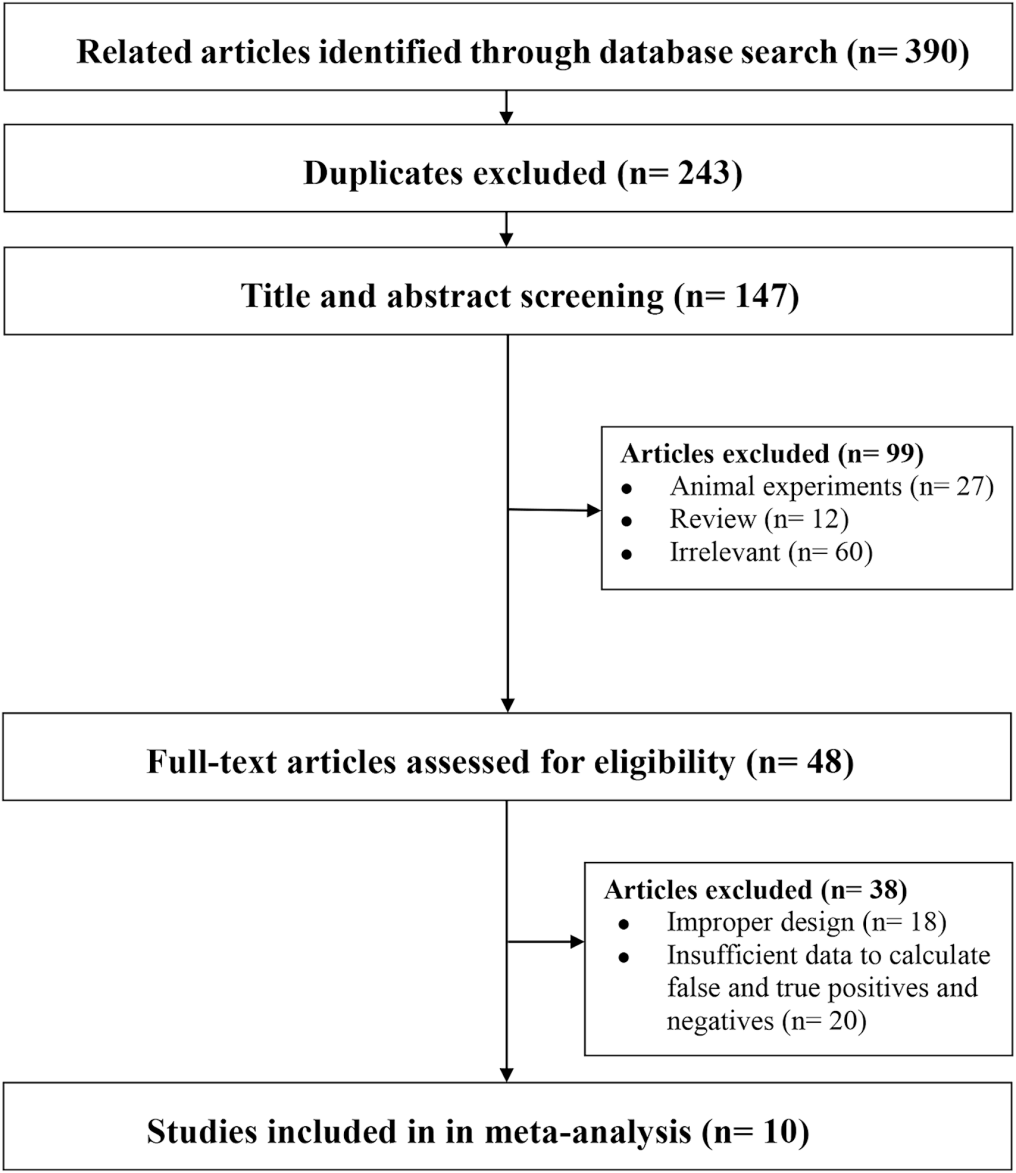
Flowchart summarizing the study selection process.

We identified 390 articles by searching the databases, of which 123 (duplicate articles), 209 (after reviewing the titles and abstracts), and 48 (after a full-text review) studies were excluded, leaving 10 studies for inclusion in our analysis (Figure 1).

### 3.2 Characteristics of the included studies

A total of 197 patients with UWS/*VS* (110 males, 87 females; mean age ranging from 35 to 52 years) and 180 patients with MCS (93 males, 87 females; mean age ranging from 37 to 53 years) were included in the analysis. As shown in Table 1, seven studies included in the research utilized task-based fMRI, while the remaining three employed resting-state fMRI. Among the task-based fMRI studies, paradigms involved mental imagery, speech processing, and hand raising. Additionally, the etiology of DoC in the included studies was diverse, mainly categorized into traumatic brain injury (TBI) and non-traumatic causes. Non-traumatic causes comprised hypoxic encephalopathy, infection, hemorrhage, stroke, and others. All included studies utilized the Coma Recovery Scale-Revised (CRS-R) as the diagnostic criteria. Finally, the levels of accuracy (range, 0.50–0.91), sensitivity (range, 0.50–0.89), and specificity (range, 0.20–0.97) of fMRI varied, indicating that the diagnostic value of fMRI for distinguishing UWS/*VS* and MCS requires further pooled analysis.

**Table 1:** Characteristics of the included studies.

Author	Year	Paradigm/method	Etiology	Diagnostic criteria	UWS/ *VS*			MCS			SEN	SPE	ACC
					*N*	Age (mean ± SD), y	Sex (F/M)	N	Age (Mean ± SD), y	Sex (F/M)			
Stender et al. (2014)	2014	Mental imagery	TBI or non-traumatic causes	CRS–R	28	43.00 ± 18.00	18/23	42	39.00 ± 17.00	29/52	0.89	0.45	0.63
Vogel et al. (2013)	2013	Mental imagery	TBI, hypoxic encephalopathy, subarachnoid, intracerebral hemorrhage, or stroke	CRS–R	10	48.00 ± 16.40	4/6	12	44.08 ± 19.20	6/6	0.50	0.75	0.64
Pipemo et al. (2012)	2012	Speech processing	TBI, hypoxic, or infection	CRS–R	19	35.00	5/14	5	37.00	1/4	0.58	0.20	0.50
Demertzi et al. (2014)	2015	Resting state	Anoxic, cerebrovascular accident, hemorrhage, seizure, or metabolic, traumatic	CRS–R	24	50.00 ± 18.00	20/4	24	50.00 ± 18.00	20/4	0.78	0.83	0.81
Wang et al. (2019)	2019	Hand raising	TBI, CVA, or anoxic brain injury	CRS–R	21	-	4/17	8	-	2/6	0.62	0.63	0.62
Yu et al. (2021)	2021	Resting State	TBI, cerebrovascular accidents, or anoxic encephalopathy	CRS–R	19	45.05 ± 16.95	9/10	19	53.84 ± 16.47	7/12	0.84	0.89	0.87
Coleman et al. (2007)	2007	Speech processing	TBI, cerebrovascular accidents, or anoxic encephalopathy	CRS–R	7	43.00	3/4	5	47.40	1/4	0.43	0.60	0.50
Kotchoubey et al. (2014)	2014	Speech processing	Anoxia, hemorrhage, or TBI	CRS–R	29	47.00 ± 14.90	11/18	26	51.00 ± 15.20	12/14	0.76	0.81	0.78
Crone et al. (2011)	2011	Resting state	Hypoxic, or TBI	CRS–R	17	52.00	5/12	8	48.00	1/7	0.65	0.75	0.68
Monti et al. (2010)	2010	Mental imagery	Anoxic brain injury, TBI, meningitis, or brain-stem stroke	CRS–R	23	43.13	8/15	31	38.13	8/23	0.83	0.97	0.91

TBI, Traumatic brain injury; CRS-R, Coma recovery scale-revised; UWS/VS, Unresponsive wakefulness syndrome/vegetative state; MCS, Minimally conscious state; N, Number of people; y, year; F, Female; M, Male; SEN, Sensitivity; SPE, Specificity; ACC, Accuracy.

### 3.3 Quality results of included studies

**Figure 2:**
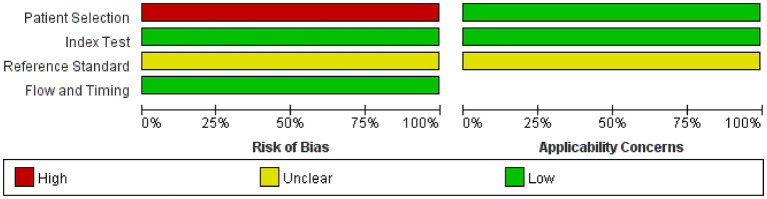
Graph of risk of bias and applicability concerns. Judgments on each area are expressed as a percentage of included studies. It consists of four parts: patient selection, index test, reference standard, and flow and timing. Bias risk was judged as “green: low,” “red: high,” or “yellow: unclear.” If the answer to all questions in a section is “yes,” then the risk of bias can be judged to be low. If the answer to any of these questions is “no,” there may be bias. The “unclear” category should only be used if the reported data is insufficient to make a judgment. Applicability sections are structured in a way similar to that of the bias sections but do not include flow and timing. Concerns about applicability are also rated as “low,” “high,” or “unclear.” The corresponding questions are in the QUADAS-2 checklist (Appendix 3).

**Figure 3:**
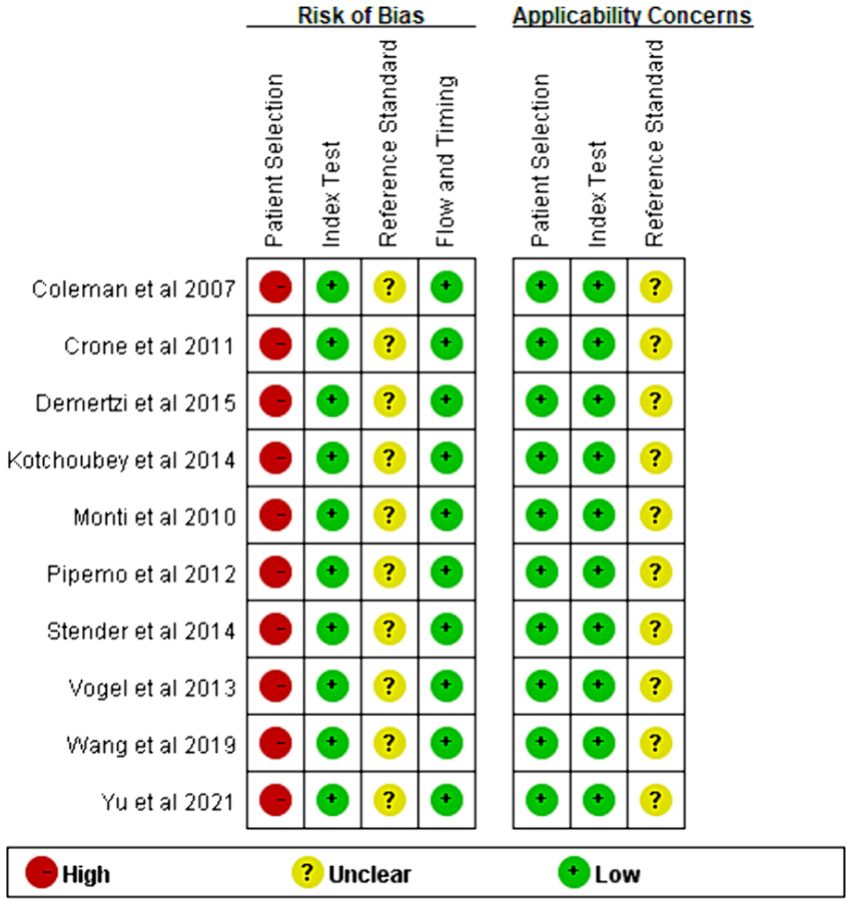
Summary of risk of bias and applicability concerns. Judgments for each area included in the study. The method is similar to Figure 2.

The risk of bias and applicability concerns for the included studies are shown in Figures 2, 3. All four parts were evaluated in terms of risk bias, and the former three parts were evaluated in terms of clinical applicability. High-risk items were mainly reflected in the patient selection part because the included studies were case–control studies rather than randomized controlled trials, and it is not clear whether the sample of patients enrolled is a consecutive case. At present, differentiation between UWS/*VS* and MCS mainly relies on bedside behavioral measurements. This lacks objectivity and uniformity, so the reference standard part was classified as an unclear risk item, and clinical applicability was classified as an unclear concern. The rest were at low risk and had minor concerns.

### 3.4 Main results

**Figure 4:**
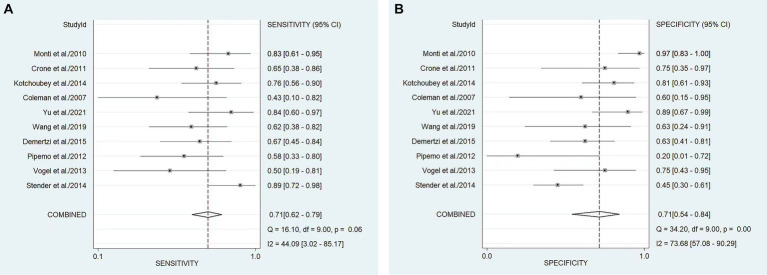
Forest plots of the sensitivity **(A)** and specificity **(B)** of the diagnostic value of fMRI for distinguishing between UWS/*VS* and MCS. The dots correspond to the individual studies included in this analysis, and both sides of the line represent the 95% confidence interval. The narrower the line is, the greater the accuracy of the study and the greater the weight. The diamond corresponds to the pooled result. The intermediate vertical line represents an invalid line.

**Figure 5:**
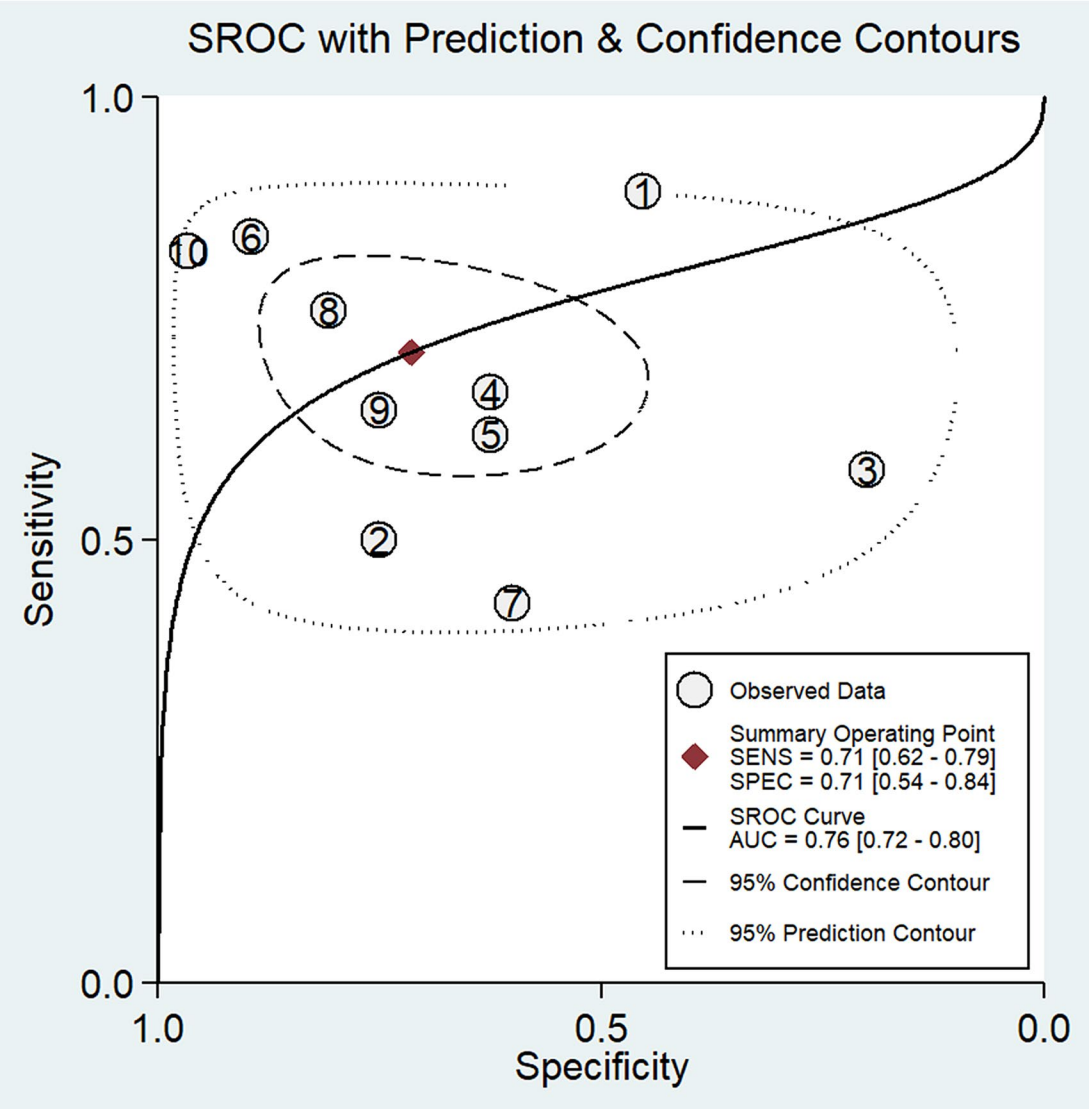
SROC plots of the diagnostic value of fMRI for distinguishing between UWS/*VS* and MCS. The ellipse shows the 95% CI for each estimate. Numbers correspond to enrolled studies as follows: 1 = Stender et al. (2014), 2 = Vogel et al. (2013), 3 = Pipemo et al. (2012), 4 = Demertzi et al. (2014), 5 = Wang et al. (2019), 6 = Yu et al. (2021), 7 = Coleman et al. (2007), 8 = Kotchoubey et al. (2014), 9 = Crone et al. (2011), and 10 = Monti et al. (2010).

**Figure 6:**
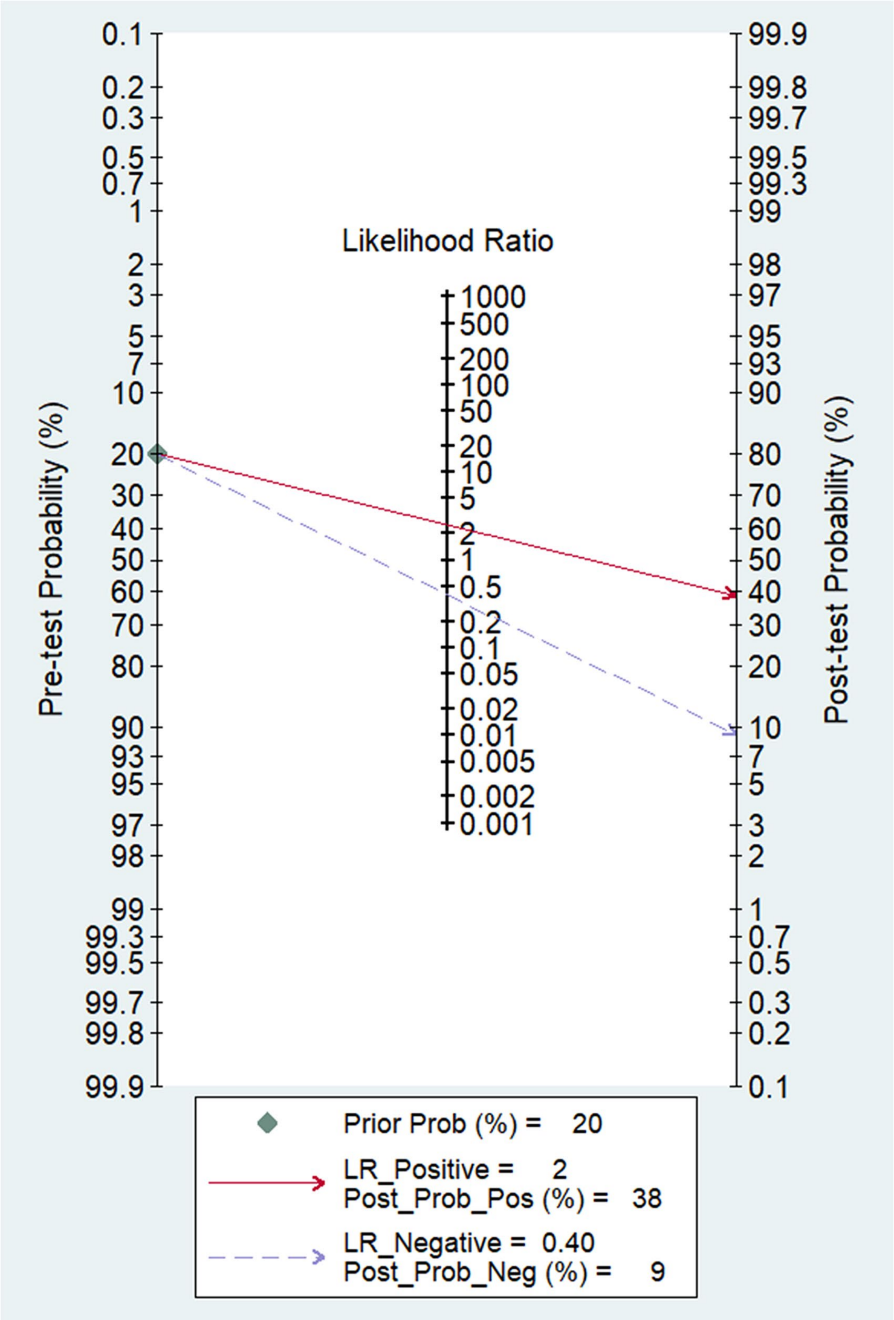
Fagan nomogram of the diagnostic value of fMRI for distinguishing between UWS/*VS* and MCS.

In our study, the pooled sensitivity and specificity of fMRI for distinguishing UWS/*VS* and MCS were 0.71 (95% CI 0.62–0.79) and 0.71 (95% CI 0.54–0.84), respectively (Figure 4). SROC curve showed that AUC was 0.76 (Figure 5). Based on these findings, the fMRI has relatively good diagnostic value for distinguishing UWS/*VS* and MCS. As shown in Figure 6, the fMRI has a moderate positive likelihood ratio (2.5) and a relatively low negative likelihood ratio (0.40), indicating that fMRI had a good ability to confirm or exclude UWS/*VS* and MCS.

### 3.5 Publication bias

**Figure 7:**
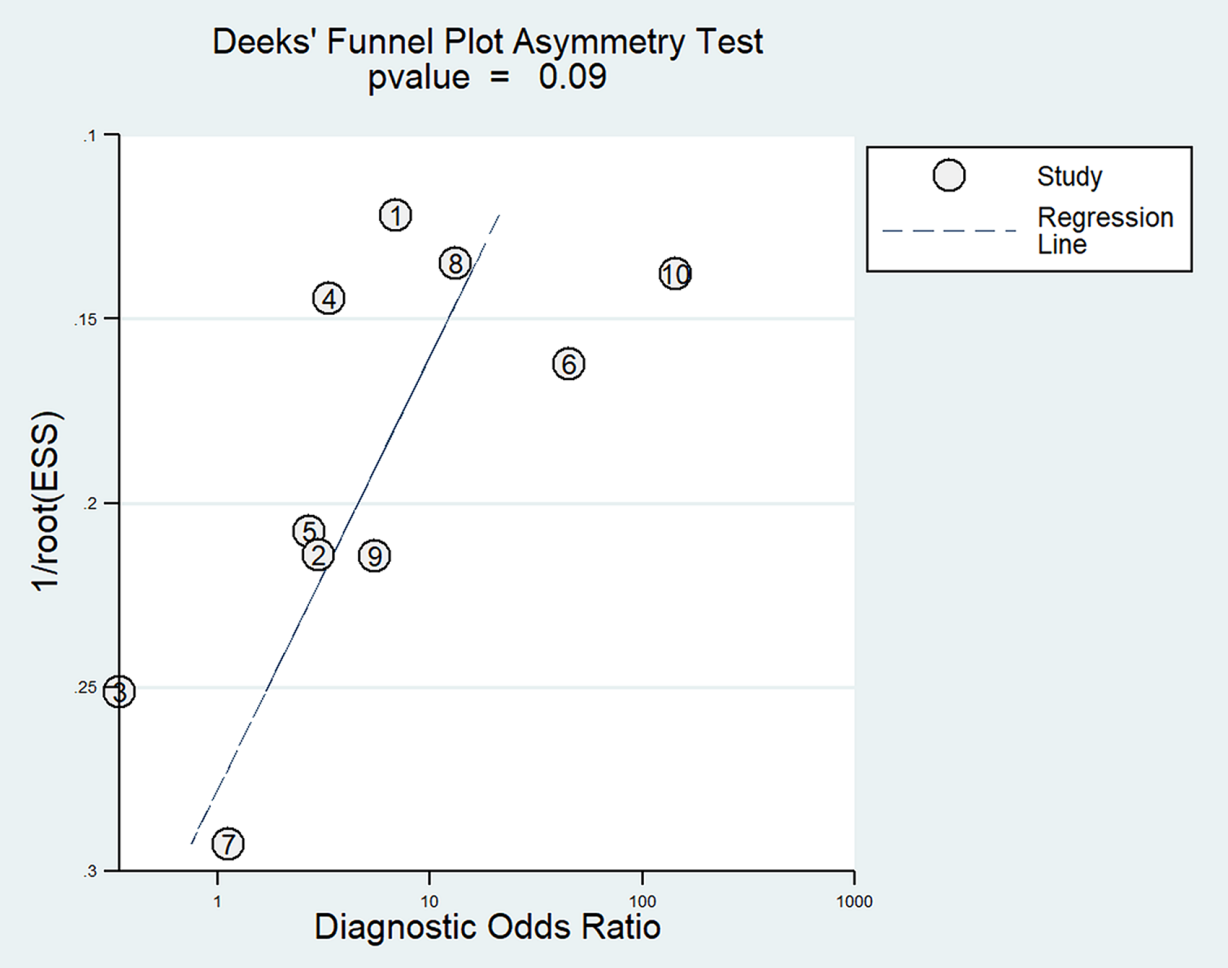
Deeks’ funnel plot asymmetry test for publication bias. Numbers correspond to enrolled studies as follows: 1 = Stender et al. (2014), 2 = Vogel et al. (2013), 3 = Pipemo et al. (2012), 4 = Demertzi et al. (2014), 5 = Wang et al. (2019), 6 = Yu et al. (2021), 7 = Coleman et al. (2007), 8 = Kotchoubey et al. (2014), 9 = Crone et al. (2011), and 10 = Monti et al. (2010). ESS, Effective sample sizes.

We determined publication bias by performing Deeks’ regression test of asymmetry (*t* = −1.90; *p* = 0.09) (Figure 7), suggesting that the statistical significance of funnel plot asymmetry is not observed, implying a relatively low likelihood of unpublished bias (*p* > 0.05).

### 3.6 Heterogeneity and meta-regression analyses

Small heterogeneity was detected among the studies (*I*^2^ = 44.00, 95% CI 30.00–85.00, *p* = 0.06). Heterogeneity was considered when *p* was less than 0.05. If *I*^2^ < 25%, no heterogeneity was noted. If the value of *I*^2^ was between 25 and 50%, the degree of heterogeneity was considered to be small. If the value of *I*^2^ was between 50 and 75% (Wu et al., 2018), heterogeneity was noted. If *I*^2^ > 75%, large heterogeneity was noted.

## 4 Discussion

As of now, there have been several reviews documenting the application developments of various neuroimaging and electrophysiological techniques in differentiating UWS/*VS* and MCS (Wu et al., 2018; Porcaro et al., 2022; Bender et al., 2015). These reviews primarily focus on elaborating different technologies, including PET, fMRI, EEG and transcranial magnetic stimulation (TMS), highlighting their respective advantages and disadvantages. Additionally, there are also relevant meta-analytic reports available. For instance, Schnakers et al. (2020) conducted a meta-analysis to explore the relationship between patients with DoC and clinical demographic variables in terms of their response to active paradigms. The study revealed that patients with MCS exhibited more favorable responses to active paradigms compared to patients with UWS/*VS*, and responders were more likely to be patients with traumatic brain injuries. Further analyses indicated that patients with the minimally conscious state minus (MCS−) and UWS/*VS* exhibit a similar likelihood of response to active paradigms. Moreover, Hannawi et al. (2015) conducted a systematic review and coordinate-based meta-analysis of studies published up to May 2014, evaluating functional neuroimaging data (fMRI, PET, and SPECT) of DoC patients in a resting state. The findings showed a significant reduction in the default mode network (DMN). The objectives of the aforementioned two meta-analyses are to investigate DoC patients’ responses to active paradigms and evaluate fMRI data of DoC patients in a resting state, which was significant for guiding clinical practice and treatment strategies. However, there has not been a summarized quantitative analysis of the sensitivity, specificity, and accuracy of these technologies in distinguishing between UWS/*VS* and MCS. In contrast, the purpose of this paper is to assess the accuracy of fMRI technology in distinguishing between UWS/*VS* and the MCS through meta-analysis, filling a gap in quantitatively analyzing using fMRI technology in distinguishing between them and providing crucial information for clinical practice. To the best of our knowledge, this is the first meta-analysis in existing research to explore the accuracy of fMRI in the discrimination and diagnosis of UWS/*VS* and MCS. Our meta-analysis results indicate that fMRI exhibits relatively good sensitivity and specificity in distinguishing between UWS/*VS* and MCS. The combined sensitivity was 0.71, specificity was 0.71, positive likelihood ratio was 2.5, negative likelihood ratio was 0.40, and the area under the SROC curve was 0.76. These findings indicate that fMRI exhibits relatively good capabilities in confirming or excluding UWS/*VS* and MCS. This discovery provides substantial support for the application of fMRI in the field of neuroimaging, particularly in dealing with the differentiation of UWS/*VS* and MCS.

In the studies included in our research, seven utilized task-based fMRI (Vogel et al., 2013; Stender et al., 2014; Pipemo et al., 2012; Coleman et al., 2007; Kotchoubey et al., 2014; Monti et al., 2010; Thibaut et al., 2021). Monti et al. (2010), using the same technology as Owen et al. (2006), discovered that among 24 patients in a vegetative state, four were conscious and able to reliably perform these tasks in fMRI. These findings demonstrate that there is a subset of patients who meet all behavioral criteria for a vegetative state but still maintain a covert level of consciousness, which cannot be detected through behavioral assessments. This has significant ethical and practical implications for both patients and their caregivers. In our included studies, Vogel et al. (2013), Stender et al. (2014), and Monti et al. (2010) also utilized the mental imagery paradigm and obtained similar findings. Moreover, Pipemo et al. (2012), Coleman et al. (2007), and Kotchoubey et al. (2014) employed the speech processing paradigm using language, music, and similar stimuli to induce activation of residual brain functions in the included studies, and it also demonstrated positive results.

In addition to using task-based fMRI, in some of the studies included, rs-fMRI has also been used to assess individuals with DoC, with the Default Mode Network (DMN) being one of the extensively studied networks, comprising the prefrontal cortex, posterior cingulate/precuneus, superior temporal cortex, hippocampus, and parietal cortex. Demertzi et al. (2014), Yu et al. (2021), and Crone et al. (2011) indicated differences in the activity patterns of DMN between UWS/*VS* and MCS individuals, and the weakened connectivity of DMN is exponentially correlated with the degree of consciousness loss, potentially serving as a crucial factor in distinguishing between UWS/*VS* and MCS. Apart from DMN, several other RSNs, potentially representing biomarkers for differences between UWS/*VS* and MCS, have been identified. These include the salience network (SN: involving the prefrontal cortex, anterior cingulate, and anterior insular circuit), the dorsal attention network (DAN: involving the insular cortex and posterior parietal), the auditory network (AN: involving the temporal cortex), the sensorimotor network (SMN: involving the striatum and parietal cortex), and the visual network (VN: involving the occipital cortex). Additionally, in the studies included, Demertzi et al. (2014) used machine learning to compare the abilities of different RSNs in distinguishing between UWS/*VS* and MCS. The results showed that differences in all the mentioned networks could distinguish between the two states with at least 80% accuracy. Among them, the auditory network provided the highest accuracy. These encouraging results emphasize the potential of rs-fMRI combined with machine learning as a highly accurate diagnostic tool for DoC.

Recently, some researchers (Noirhomme et al., 2010) have proposed that the state of consciousness is associated with connectivity between multiple brain regions, particularly involving thalamocortical connections within the frontoparietal network. Functional neuroimaging has revealed widespread metabolic dysfunctions in patients with DoC, specifically within the frontal and parietal lobes known as the “global neural workspace.” This encompasses both the midline default mode network (DMN) and the lateral cortices of these lobes referred to as the “intrinsic” and “extrinsic” systems, respectively. Zhou (2017) found consistent reductions in neuronal activity in the thalamus and frontal/temporal regions using fractional amplitude of low-frequency fluctuations (fALFF) and functional connectivity MRI based on resting-state and task-related fMRI even in patients with mild traumatic brain injury. White and Alkire (2003) also discovered that anesthesia-induced loss of consciousness correlates with decreased cortico-cortical and thalamocortical connectivity within both intrinsic and extrinsic networks. Collectively, these findings suggest a close association between conscious awareness and functional integrity of thalamocortical and cortico-cortical connections across different brain regions.

While fMRI demonstrates potential advantages in distinguishing between UWS/*VS* and MCS, it is noteworthy that in some of the included studies, we observed a relatively high false-negative rate for fMRI. This may be attributed to the fact that patients in an MCS may not comprehend tasks or lack motivation to participate. Therefore, neuroimaging diagnosis should not exist in isolation and should be complemented by the joint application of behavioral assessments and/or other examination techniques. For instance, combining EEG and/or PET can offer more comprehensive information about brain activity, aiding in a more accurate understanding of the brain functional status in patients with UWS/*VS* and MCS (Formaggio et al., 2020; Raveendran et al., 2024; Boly et al., 2008). These multimodal fusion approaches are expected to provide a more comprehensive insight into the neural mechanisms of UWS/*VS* and MCS, promoting advances in clinical management and decision-making. Moreover, fMRI technology itself has some limitations, such as individual differences in patients, temporal changes in the disease course, discomfort during the scanning process, and common motion artifacts in individuals with UWS/VS. These factors may restrict the feasibility of fMRI in bedside clinical applications. Future research should focus on standardized study designs and larger sample sizes to further validate the reliability and effectiveness of fMRI in this field. Simultaneously, enhancing the training of healthcare professionals to increase awareness and understanding of this technology will contribute to the practical application of fMRI in the management of individuals with UWS/VS. We can anticipate more studies exploring the application of fMRI in various clinical contexts. For example, can monitor the brain activation patterns of individuals with UWS/*VS* in fMRI predict their potential for recovery? This predictive neuroimaging could have significant clinical applications in rehabilitation medicine, facilitating the development of individualized rehabilitation plans.

Through Deeks’ regression test, no significant publication bias was observed in the included studies, indicating the reliability of the summarized results. Additionally, the I^2^ test result showed *I*^2^ = 44%, falling between 25 and 50%, suggesting a relatively low degree of heterogeneity among the studies. Finally, an assessment of bias and applicability issues for the included studies was conducted using the QUADAS-2 checklist. The majority of items were rated as low risk and low concern. However, there were high-risk items in terms of patient selection, as some studies used case–control designs rather than randomized controlled trials.

Our study has some limitations. Firstly, our meta-analysis focused primarily on the diagnostic value of fMRI in patients with DoC, and further meta-analyses are needed for the diagnostic value of EEG or PET-CT in patients with chronic consciousness disorders. Secondly, we only included studies written in English. Additionally, considering the integration of fMRI with other neuroimaging techniques may establish a more comprehensive, multimodal assessment system, providing more accurate diagnoses and personalized medical management for patients.

## 5 Conclusion

This study conducted a meta-analysis to explore the accuracy of fMRI in the discrimination and diagnosis of UWS/*VS* and MCS. Our meta-analysis indicated that this technology holds potential diagnostic value in distinguishing between UWS/*VS* and MCS. Future research should place greater emphasis on standardized study designs and include larger sample sizes to further validate the reliability and effectiveness of fMRI in this field.

## Data Availability

The original contributions presented in the study are included in the article/Supplementary material; further inquiries can be directed to the corresponding author.
