# Supplementary material for: Meta-analysis of the diagnostic value of functional magnetic resonance imaging for distinguishing unresponsive wakefulness syndrome/vegetative state and minimally conscious state
Source: Front Neurosci. 2024 Sep 9;18:1395639. doi: 10.3389/fnins.2024.1395639 (PMC11417101; doi:10.3389/fnins.2024.1395639)
Supplement: Supplementary file 1 [file Data_Sheet_1.docx]

**
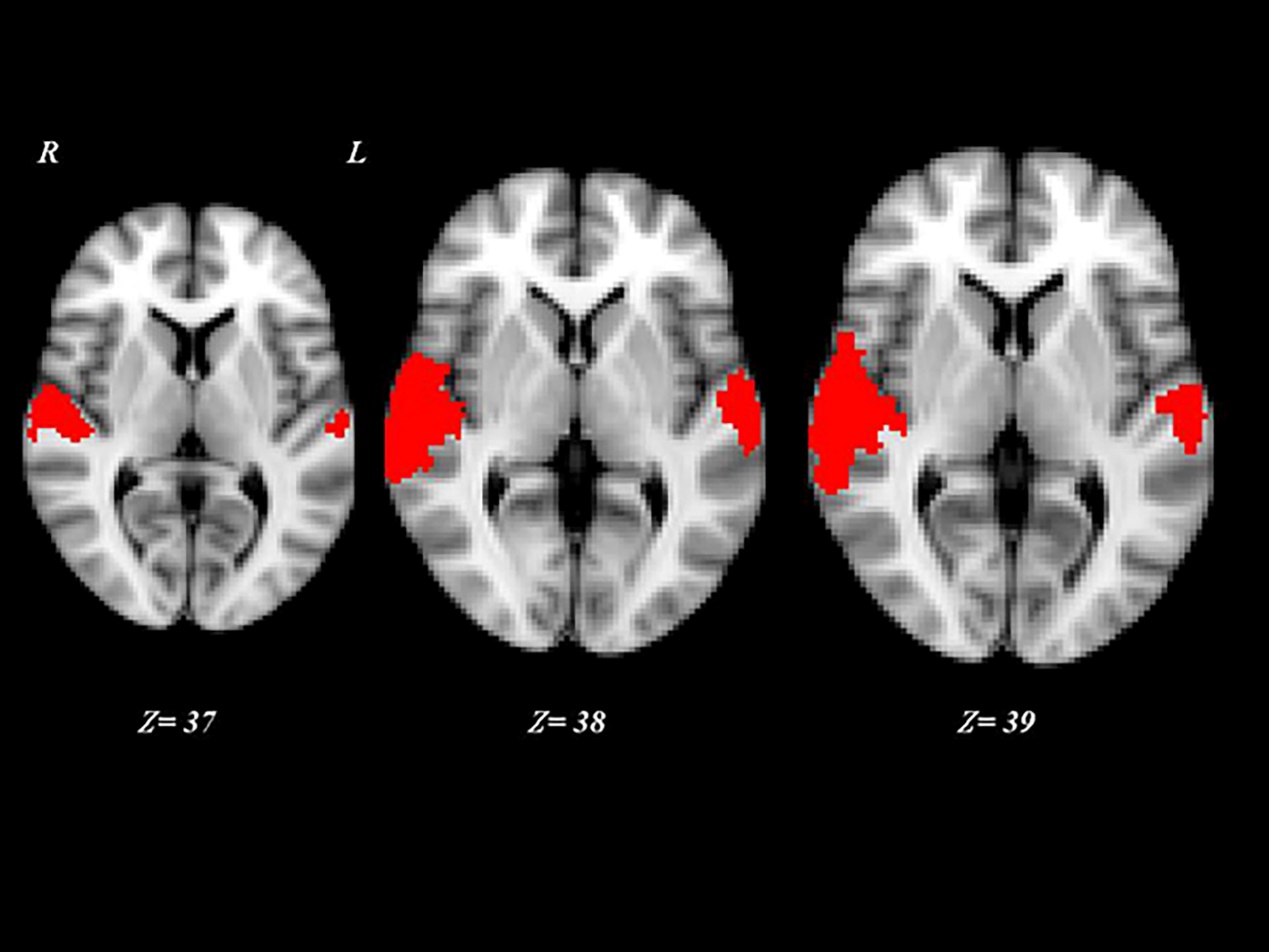
**

**Figure S1.** Post-hoc contrasts where both “VS converted” were compared with MCS and “VS converted”. Red shows activated brain areas (p<0.01, cluster corrected) as results of a subtraction analysis among activation VS stable, MCS and VS converted patients. Z coordinates are expressed in mm.

**Source from**: Marino S, Bonanno L, Ciurleo R, Baglieri A, Morabito R, Guerrera S, et al. Functional Evaluation of Awareness in Vegetative and Minimally Conscious State. Open Neuroimag J. 2017;11:17-25.

**Copyright permission:**

**
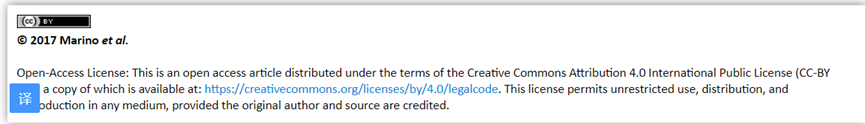
**

**
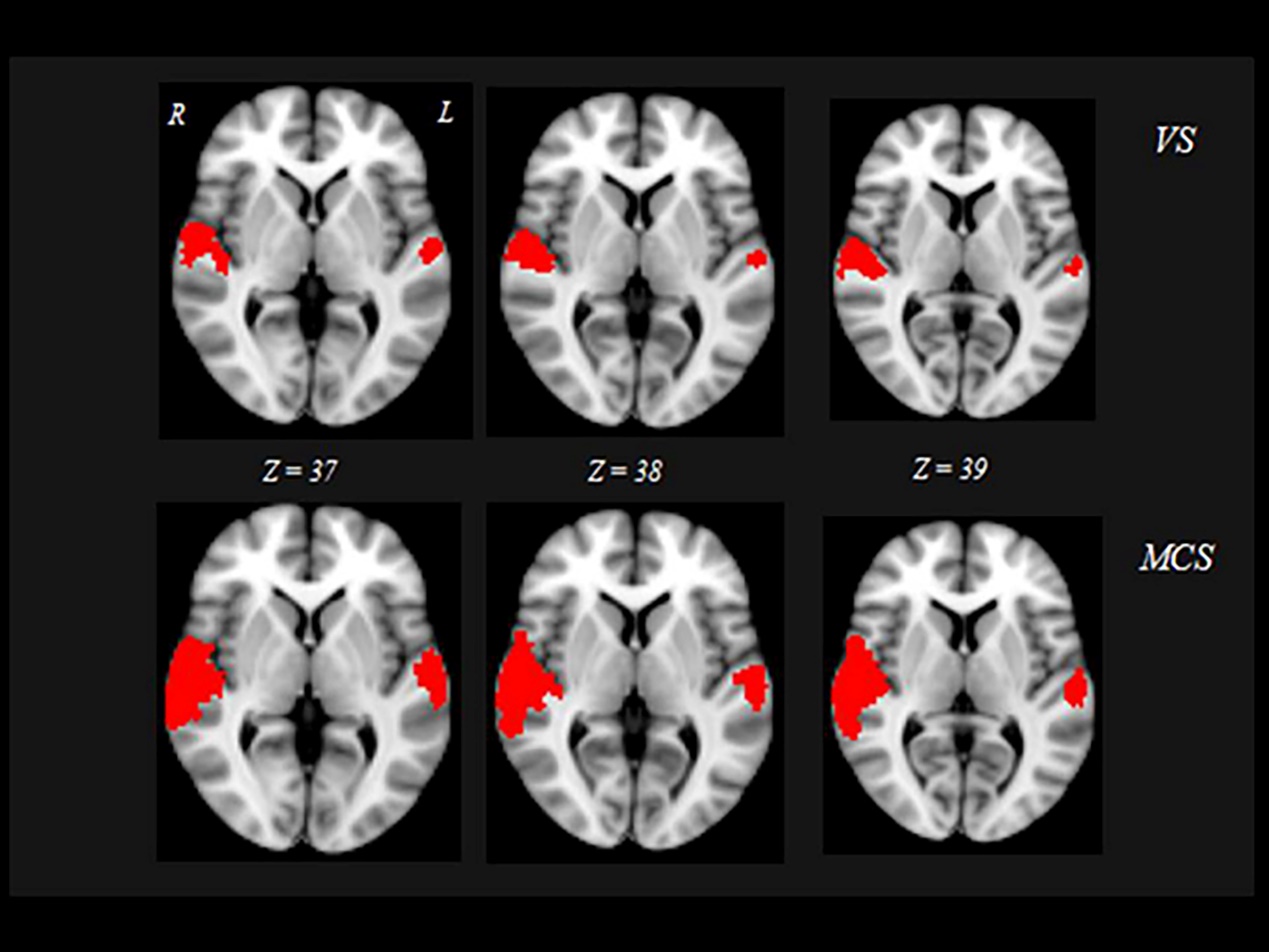
**

**Figure S2.** Red shows activated brain areas (p<0.01, cluster corrected for multiple comparisons) during acoustic stimuli in “VS stable”, and MCS patients, overlaid on the MNI standard brain. Z coordinates are expressed in mm.

**Source from**: Marino S, Bonanno L, Ciurleo R, Baglieri A, Morabito R, Guerrera S, et al. Functional Evaluation of Awareness in Vegetative and Minimally Conscious State. Open Neuroimag J. 2017;11:17-25.

**Copyright permission:**


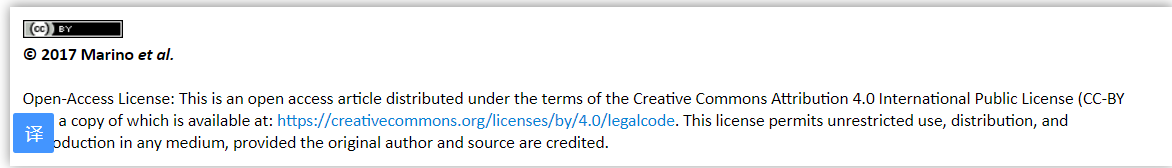


**
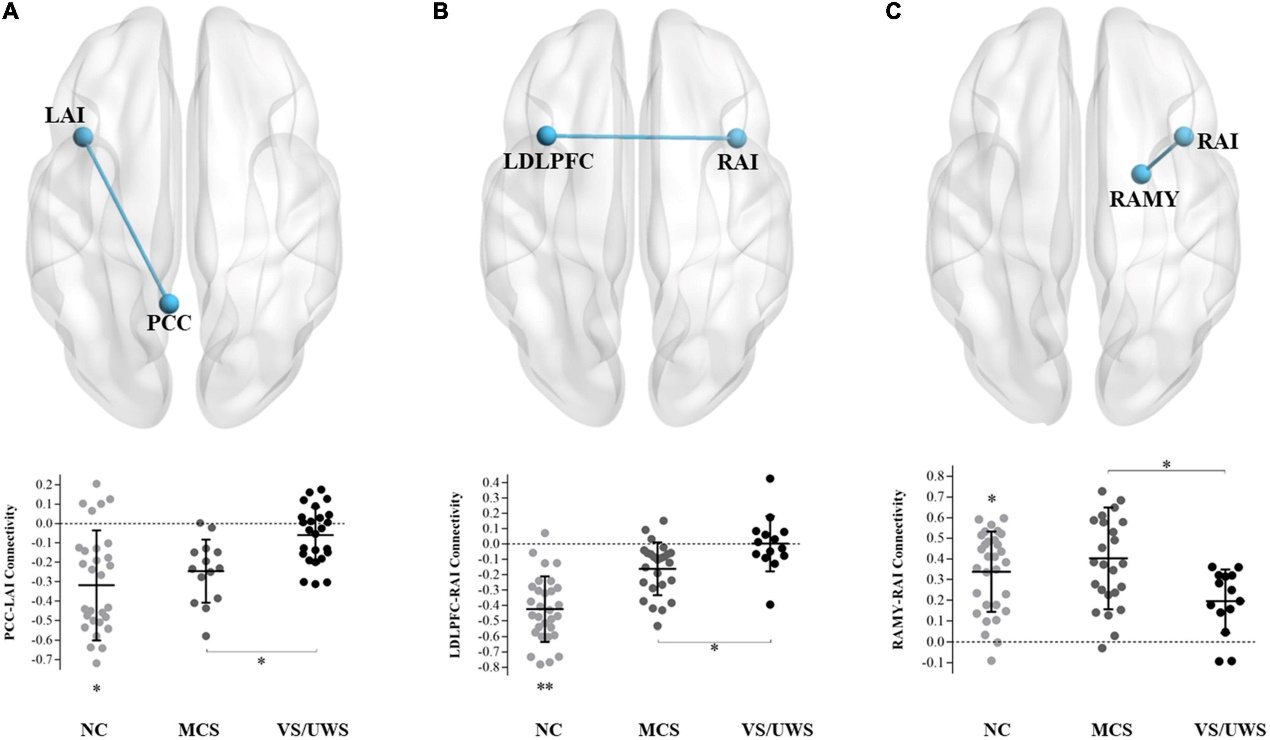
**

**Figure S3.** Connectivity differences between MCS and VS/UWS patient groups and NC group in ROI-wise analyses. Between-group comparisons of the ROI-wise analyses indicated significantly decreased functional connectivity between PCC-LAI (A), RAI-LDLPFC (B), and RAI-RAMY (C) during rest, in the VS/UWS group relative to MCS and NC groups. The graphs illustrate the differences in functional connectivity of PCC-LAI, RAI-LDLPFC, and RAI-RAMY in MCS, VS/UWS patients and NC groups (AI, anterior insula; PCC, posterior cingulate cortex; DLPFC, dorsolateral prefrontal cortex; AMY, amygdala; L, left; R, right; NC, healthy control individuals; MCS, minimally conscious state; VS/UWS, vegetative-state/unresponsive wakefulness syndrome; *p < 0.05; **p < 0.01; error bars represent the standard error of measurement).

**Source from**: Wang YT, Chen SS, Xia XY, Peng Y, Wu B. Altered functional connectivity and regional brain activity in a triple-network model in minimally conscious state and vegetative-state/unresponsive wakefulness syndrome patients: A resting-state functional magnetic resonance imaging study. Frontiers in Behavioral Neuroscience. 2022;16.

**Copyright permission:**

**
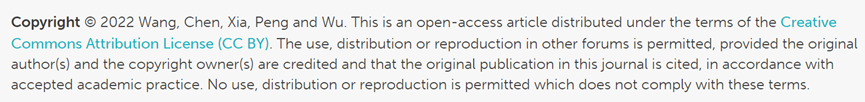
**
