# Supplementary material for: Meta-analysis of the diagnostic value of functional magnetic resonance imaging for distinguishing unresponsive wakefulness syndrome/vegetative state and minimally conscious state
Source: Front Neurosci. 2024 Sep 9;18:1395639. doi: 10.3389/fnins.2024.1395639 (PMC11417101; doi:10.3389/fnins.2024.1395639)
Supplement: Supplementary file 2 [file Data_Sheet_2.pdf]

Pubmed search strategy:

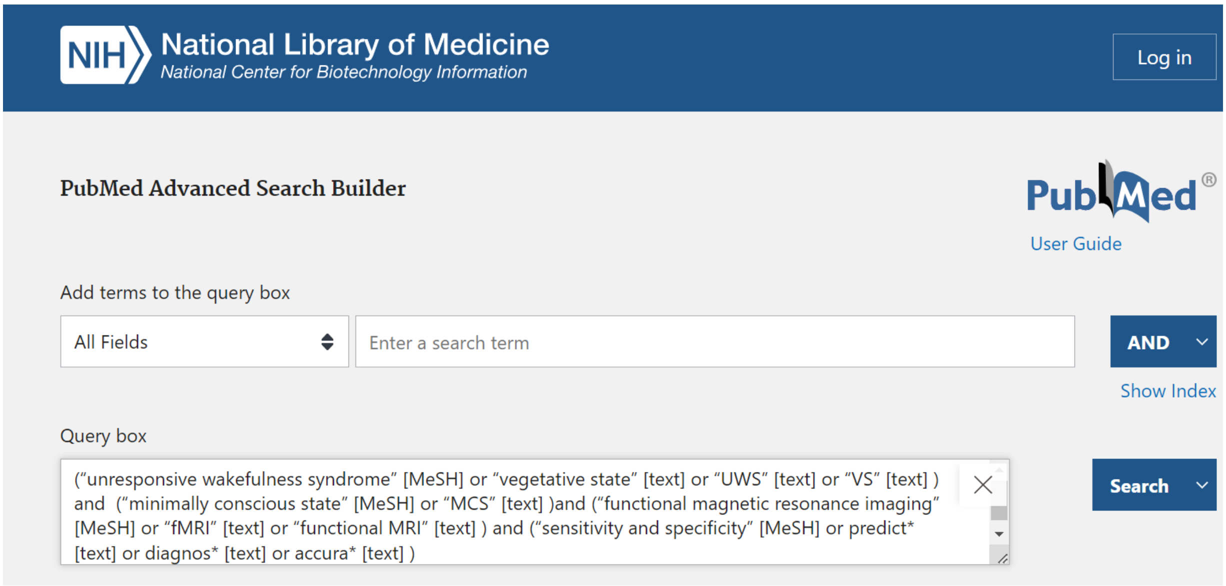

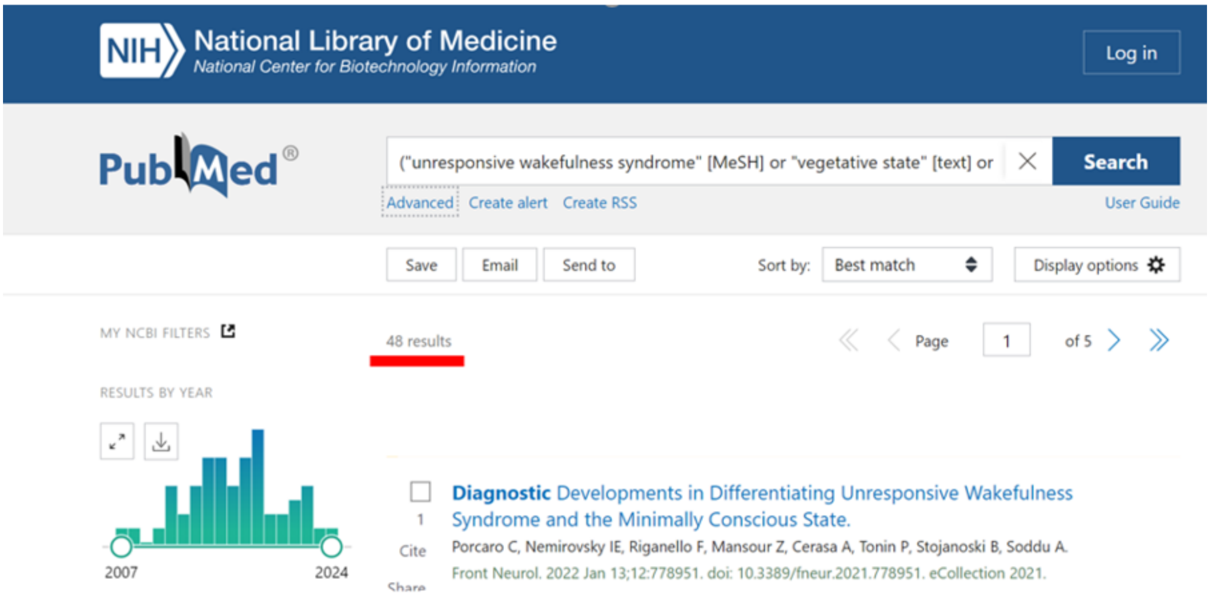

Number of articles: 48
