# Supplementary material for: Meta-analysis of the diagnostic value of functional magnetic resonance imaging for distinguishing unresponsive wakefulness syndrome/vegetative state and minimally conscious state
Source: Front Neurosci. 2024 Sep 9;18:1395639. doi: 10.3389/fnins.2024.1395639 (PMC11417101; doi:10.3389/fnins.2024.1395639)
Supplement: Supplementary file 3 [file Data_Sheet_3.docx]

| **Author** | **Year** | **fMRI details** |
| --- | --- | --- |
| Stender et al.[19] | 2014 | Not mentioned |
| Vogel et al.[20] | 2013 | The fMRI was performed on a 1.5-T magnetic resonance imaging scanner. a Changes in the BOLD T2*-weighted magnetic resonance signal were measured using a gradient echo-planar imaging sequence (repetition time[TR]Z3410ms; echo delay time [TE]Z50ms; field of view [FoV]Z192mm; flip angleZ90°; 64 × 64 matrix; 36 slices covering the whole brain; slice thickness, 3.0mm; no gap; voxel size 3 ×3×3mm). A T1-weighted anatomical image was obtained from each subject to allow anatomical localization (TRZ2300ms; TEZ2.98ms; 160 slices; voxel size1.0 × 1.0 ×1.1mm). Image processing and statistical analysis were conducted with Statistical Parametric Mapping version 8. ^24,b^ Preprocessing included realignment, coregistration, segmentation, and spatial normalization (template of the Montreal Neurological Institute). Thereafter, a Gaussian filter of 8-mm full width at half maximum was applied to smooth the data spatially. |
| Pipemo et al.[21] | 2012 | A General Electric (Milwaukee, WI, USA) 3 Tesla Signa Excite system and an eight-chan-nel phased array coil were used to acquire fMRI and morphological sequences. The following parameters were used. fMRI. A whole brain, 29 interleaved slices, axial (bicommissural AC-PC plane), single-shot, gradient-echo echo-planar (GE-EPI) sequence was continuously acquired during the stimulation with the following parameters: slice thickness 4.0 mm, gap 0.0 mm, field of view 24 cm, number of excitations 1.0, flip angle 90° and a total acquisition time of 5 min 18s (18 s of dummy scans, 6 volumes). Structural MRI. A whole brain (166 slices) high definition isovolumetric sagittal spoiled gradient recall- inversion recovery (SPGR-IR) sequence was acquired, with the following parameters: repetition time 7.3 ms, echo time 3.2 ms, matrix 288 ×288, slice thickness 1.0 mm, gap 0.0 mm, field of view 29 cm, number of excitations 1.0, flip angle 10° and a total acquisition time of 6 min 35s. |
| Demertzi et al. [22] | 2015 | In all patients and controls, functional MRI time series were acquired on a 3 T head-only scanner (Siemens Trio, Siemens Medical Solutions, Erlangen, Germany) operated with a standard transmit-receive quadrate head coil. Three hundred multislice T2*-weighted functional images were acquired with a gradient-echo echo-planar imaging sequence using axial slice orientation and covering the whole brain (32 slices; voxel size: 3 × 3 × 3 mm3 ; matrix size 64 × 64× 32; repetition time ¼ 2,000 msec; echo time ¼ 30 msec; flip angle ¼ 78 ; field of view ¼ 192 × 192 mm 2 ). The three initial volumes were discarded to avoid T1 saturation effects. For anatomical reference, a high-resolution T1- weighted image was acquired for each subject (T1-weighted 3D magnetization-prepared rapid gradient echo sequence). |
| Wang et al. [23] | 2019 | Data were collected using a 1.5T General Electric Sigma Horizon MRI system (GE Medical Systems, Milwaukee, WI, United States). First, 22 axial anatomic images were collected using a T1-weighted spin echo sequence [repetition time (TR) = 500 ms, echo time (TE) = 9 ms, field of view (FOV) = 240 × 240 mm, slice thickness = 5 mm, skip = 1 mm, matrix = 256 × 256, with the resolution of three dimensions of one voxel: x = 0.9375 mm, y = 0.9375 mm, z = 6 mm]. Next, 120 images per slice were acquired using a gradient echo planar imaging (TR = 3000 ms, TE = 60 ms, matrix = 64 × 64, with the resolution of three dimensions of one voxel: x = 3.75 mm, y = 3.75 mm, z = 6 mm). Finally, a fast spoiled gradient recalled sequence (TR = 27 ms, TE = 6 ms, FOV = 240 × 240 mm, matrix = 256 × 256, with the resolution of three dimensions of one voxel: x = 1.3 mm, y = 0.9375 mm, z = 0.9375 mm) was used in a sagittal plane to collect three-dimensional images covering the entire brain volume. |
| Yu et al. [24] | 2021 | All the subjects underwent MRI scans on a Magnetom Essenza 1.5 Tesla scanner (Siemens, Germany) with a homogeneous birdcage head coil. High-resolution anatomical images were acquired with a T1-weighted three-dimensional magnetization-prepared rapid gradient ehco sequence with sagittal scanning (repetition time [TR]/echo time [TE]: 2000/5.18 ms; field of view [FOV]: 240 mm ×240 mm; matrix size: 256 × 256; flip angle:15°; 160 slices; slice thickness: = 1 mm; gap: 0.5 mm). For resting state fMRI, we used a gradient echo planar imaging sequence sensitive to BOLD contrast (TR:2000; TE: 40 ms; FOV: 240 mm ? 240 mm; flip angle: 90°; matrix size: 64 × 64; slice thickness: 5 mm; gap:1 mm; phase per location: 240; scan time: 8 min; slice order: interleaved). |
| Coleman et al. [25] | 2007 | The fMRI imaging data was acquired using a Bruker Medspec (Ettlingen, Germany) 3-Tesla MR system with a head gradient set. Each volume consisted of 21?4mm thick slices with an interslice gap of 1mm; FOV: 25?25cm; matrix size, 128×128, TE=27ms; acquisition time 1.6s; actual TR=9s. Acquisition was transverse -oblique, angled away from the eyes, and covered all of the brain. In addition to the functional data, a 3D T1-weighted SPGR image with 1mm isotropic spatial resolution was acquired for each patient. |
| Kotchoubey et al. [26] | 2014 | Participants were scanned in a 1.5 T Siemens Symphony MR Scanner (Siemens, Erlangen, Germany). Functional images were acquired using a T2*-weighted echo-planar imaging (EPI) sequence with the following parameters: repetition time = 3410 ms, echo time = 50 ms, Field of View = 192 mm, flip angle = 90°, 64 x 64 matrix, 36 slices covering the whole brain, slice thickness 3 mm, no gap, voxel size 3 x 3 x 3 mm. An anatomic MRI for superimposition with functional images was acquired using a T1-weighted three-dimensional multiplanar reconstructed sequence (repetition time = 1860 ms, echo time = 3.57 ms, 176 slices, slice thickness = 1mm, voxel size 0.9 x 0.8 x 1.0 mm). The patient’s vital parameters (heart rate, oxygen saturation) were monitored by an experienced physician throughout the measurement. Head movement was mini-mized by using a form cushion. |
| Crone et al. [27] | 2011 | patients’ and controls’ fMRI data were acquired using a 1.5T scanner (Philips Gyroscan Intera) and two 3T scanners (Philips Achieva and Siemens TIM TRIO) due to hardware changes at the clinical setting. Number of control subjects and patients were matched for field strength. Six control subjects, 2 patients in MCS and 4 with UWS were scanned with the 1.5T Philips scanner. Four control subjects, 4 MCS and 10 UWS patients were scanned with the 3 T Philips scanner. For both Philips scanners, 134 T2*-weighted images were obtained with a gradient echo-planar sequence (EPI) in axial plane (25 slices with a thickness of 4.5 mm and an inter-slice gap of 0.5 mm; matrix size=64664; FoV=210 mm 2 ; TR=2200 ms; TE=45 ms; flip angle=90u). The data of the remaining 15 control subjects, 2 MCS and 3 UWS patients were acquired with the 3 T Siemens scanner. Again, 134 T2*-weighted images were obtained with a gradient EPI in axial plane (25 slices with a thickness of 4.5 mm and an inter-slice gap of 0.5 mm; matrix size=80680; FoV=210 mm 2 ; TR=2200 ms; TE=30 ms; flip angle=70u). In addition, T1-weighted MPRAGE sequences for anatomic information were acquired for each participant. |
| Monti et al [28] | 2010 | Not mentioned |

[19] Stender J,Gosseries O,Bruno MA, et al. Diagnostic precision of PET imaging and functional MRI in disorders of consciousness: a clinical validation study. Lancet. 2014;384 (9942):514-22. doi:10.1016/S0140-6736(14)60042-8

[20] Vogel D,Markl A,Yu T, et al. Can mental imagery functional magnetic resonance imaging predict recovery in patients with disorders of consciousness?. Arch Phys Med Rehabil. 2013;94 (10):1891-8. doi:10.1016/j.apmr.2012.11.053

[21] Piperno R,Battistini A,Cevolani D, et al. FMRI activation with an "affective speech" paradigm in vegetative and minimally conscious States: applicability and prognostic value. Neuroradiol J. 2012;25 (3):289-99. doi:10.1177/197140091202500303

[22] Demertzi A,Gómez F,Crone JS, et al. Multiple fMRI system-level baseline connectivity is disrupted in patients with consciousness alterations. Cortex. 2014;52:35-46. doi:10.1016/j.cortex.2013.11.005

[23] Wang F,Hu N,Hu X, et al. Detecting Brain Activity Following a Verbal Command in Patients With Disorders of Consciousness. Front Neurosci. 2019;13:976. doi:10.3389/fnins.2019.00976

[24] Yu Y,Chen S,Zhang L, et al. Disrupted Strength and Stability of Regional Brain Activity in Disorder of Consciousness Patients: A Resting-State Functional Magnetic Resonance Imaging Study. Neuroscience. 2021;469:59-67. doi:10.1016/j.neuroscience.2021.06.031

[25] Coleman MR,Rodd JM,Davis MH, et al. Do vegetative patients retain aspects of language comprehension? Evidence from fMRI. Brain. 2007;130 (Pt 10):2494-507. doi:10.1093/brain/awm170

[26] Kotchoubey B,Yu T,Mueller F, et al. True or false? Activations of language-related areas in patients with disorders of consciousness. Curr Pharm Des. 2014;20 (26):4239-47.

[27] Crone JS,Ladurner G,Höller Y, et al. Deactivation of the default mode network as a marker of impaired consciousness: an fMRI study. PLoS One. 2011;6 (10):e26373. doi:10.1371/journal.pone.0026373

[28] Monti MM,Vanhaudenhuyse A,Coleman MR, et al. Willful modulation of brain activity in disorders of consciousness. N Engl J Med. 2010;362 (7):579-89. doi:10.1056/NEJMoa0905370
