# Supplementary material for: Meta-analysis of the diagnostic value of functional magnetic resonance imaging for distinguishing unresponsive wakefulness syndrome/vegetative state and minimally conscious state
Source: Front Neurosci. 2024 Sep 9;18:1395639. doi: 10.3389/fnins.2024.1395639 (PMC11417101; doi:10.3389/fnins.2024.1395639)
Supplement: Supplementary file 4 [file Data_Sheet_4.docx]

**Quality Assessment of Diagnostic Accuracy Studies-2 (QUADAS-2) checklist**

**Part 1: Patient Selection**

Risk of Bias:

item 1: Was a consecutive or random sample of patients enrolled?

item 2: Was a case–control design avoided?

item 3: Did the study avoid inappropriate exclusions?

Applicability:

item 4: Are there concerns that the included patients and setting do not match the review question?

**Part 2: Index Test**

Risk of Bias:

item 5: Were the index test results interpreted without knowledge of the results of the reference standard?

item 6: If a threshold was used, was it prespecified?

Applicability:

item 7: Are there concerns that the index test, its conduct, or its interpretation differ from the review question?

**Part 3: Reference Standard**

Risk of Bias:

item 8: Is the reference standard likely to correctly classify the target condition?

item 9: Were the reference standard results interpreted without knowledge of the results of the index test?

Applicability:

item 10: Are there concerns that the target condition as defined by the reference standard does not match the question?

**Part 4: Flow and Timing**

Risk of Bias:

item 11: Was there an appropriate interval between the index test and reference standard?

item 12: Did all patients receive a reference standard

item 13: Did all patients receive the same reference standard?

item 14: Were all patients included in the analysis?
